# Gene-based immunotherapy in osteosarcoma: from oncolytic vectors to engineered immune cells

**DOI:** 10.3389/fimmu.2026.1920347

**Published:** 2026-08-04

**Authors:** Junliang Jia, Wei Xie, Jiawang Zhou, Weidong Wu, Shuliang Zhou

**Affiliations:** Department of Orthopedics, Suzhou Ninth People’s Hospital, Soochow University, Suzhou, Jiangsu, China

**Keywords:** clinical translation, gene therapy, immune reprogramming, immunotherapy, oncolytic vector, osteosarcoma, tumor microenvironment

## Abstract

Osteosarcoma remains difficult to cure once it metastasizes, recurs, or becomes resistant to chemotherapy. Surgery combined with multi-drug chemotherapy improves outcomes in localized disease, but outcomes for high-risk patients have plateaued, prompting renewed interest in immunotherapy. However, immune checkpoint blockade has limited efficacy in unselected osteosarcoma patients: in SARC028, one objective response was observed among 22 patients with osteosarcoma treated with pembrolizumab. This limited response is consistent with antigen heterogeneity, enrichment of immunosuppressive myeloid populations, insufficient cytotoxic lymphocyte infiltration, matrix-associated immune exclusion, and metastatic immune adaptation. Gene-based immunotherapy offers a complementary strategy. When endogenous immune priming is inadequate, these platforms may initiate, reshape, or amplify antitumor immunity by promoting oncolytic antigen release, local cytokine or chemokine expression, engineered immune-cell recognition, or non-viral delivery of immunomodulatory payloads. This article discusses oncolytic vectors, cytokine-armed vectors, CAR-T and CAR-NK cells, TCR- or neoantigen-directed strategies, and non-viral gene delivery systems as potential approaches for reshaping osteosarcoma immunity. It also examines combination strategies and translational challenges in pediatric and adolescent patients. Beyond summarizing platform-level evidence, we distinguish findings generated directly in osteosarcoma from evidence extrapolated from other tumor types, identify the principal unresolved knowledge gaps for each platform, and summarize the current osteosarcoma-specific clinical trial landscape to clarify the translational maturity of these strategies.

## 1 Introduction

Osteosarcoma is the most common primary malignant bone tumor in children and adolescents, yet advances in tumor biology have translated into only limited therapeutic benefit. Surgery combined with multi-drug chemotherapy can cure some patients with localized disease, but the prognosis remains poor for patients with lung metastases, recurrence, unresectable lesions, or chemotherapy resistance (1). Standard multi-agent chemotherapy regimens (typically methotrexate, doxorubicin, and cisplatin) have not changed substantially in more than three decades, carry considerable acute and long-term toxicity in a predominantly pediatric and adolescent population, and add little further benefit once micrometastatic disease becomes overtly resistant; this therapeutic stagnation is the practical justification for pursuing gene-based immunotherapy as a mechanistically distinct approach. Contemporary reviews still describe osteosarcoma as a disease with a treatment plateau, and intensive cytotoxic therapy rarely addresses the core issues of drug-resistant or disseminated osteosarcoma (2, 3). This clinical bottleneck has driven interest in immunotherapy, although clinical activity has so far been limited. In SARC028, 40 patients with bone sarcoma were evaluable for response: two (5%) had an objective response, consisting of one response among 22 patients with osteosarcoma and one among five with chondrosarcoma; no objective response was reported in the 13-patient Ewing sarcoma subgroup (4). Thus, osteosarcoma-specific activity was limited to one response among 22 patients, an insufficient signal to support unselected checkpoint blockade. This does not mean that osteosarcoma is immunologically irrelevant, but rather suggests that the disease often lacks the immune conditions required for standard checkpoint therapy to be effective: insufficient pre-activated effector T cells, excessive myeloid suppression, and poorly coordinated antitumor immune responses.

In this context, gene-based immunotherapy is best understood as a strategy for immune-niche reprogramming. The platforms reviewed here are considered according to the osteosarcoma barrier they are designed to address: oncolytic vectors can overcome weak antigen release and innate sensing; cytokine- or chemokine-armed vectors can strengthen local priming and effector-cell recruitment; engineered immune cells can enforce antigen recognition but must overcome trafficking, persistence, exhaustion, and antigen escape; and non-viral systems can deliver transient payloads to tumor, immune, stromal, or metastatic compartments. Because much of the mechanistic rationale for these platforms was first established in melanoma, glioma, or adult carcinomas, this review explicitly distinguishes evidence generated directly in osteosarcoma from evidence extrapolated from other tumor types, while highlighting the principal unresolved questions that should guide future osteosarcoma-focused studies throughout the review.

This Mini Review draws on English-language primary clinical studies, osteosarcoma-specific preclinical studies, and comparative canine osteosarcoma models, with recent reviews used mainly to identify platform classes and unresolved translational questions. Evidence from broader sarcoma or solid-tumor studies is included selectively when osteosarcoma-specific data are unavailable and is identified as extrapolative rather than presented as disease-specific proof. Literature published through July 2026 was considered.

## 2 Immune resistance in osteosarcoma and the rationale for gene-based therapy

The rationale for gene-based immunotherapy is grounded in the immunophenotype of osteosarcoma itself. Unlike much of the vector- and cell-engineering literature discussed in later sections, the evidence summarized in this section is drawn directly from human or canine osteosarcoma specimens rather than extrapolated from other tumor types, and therefore provides the most disease-specific rationale available. Zhou et al. constructed a single-cell RNA atlas of advanced osteosarcoma, revealing significant intratumoral heterogeneity, variable antigen presentation, and immunosuppressive cellular programs dominated by tumor-associated macrophages and osteoclasts (5). Jiang et al. further identified prognostically distinct osteosarcoma subtypes through multi-omics analysis, supporting the view that osteosarcoma is not always an immune-cold tumor, but often presents an immune-excluded phenotype, in which immune cells are present within the tumor but functionally segregated from malignant cells rather than truly absent (6). Some lesions contain immune cells, but these cells may be spatially separated from viable tumor regions, dominated by a suppressive myeloid state, or insufficient to form a durable cytotoxic response. Myeloid biology is particularly important. Tumor-associated macrophages, monocyte-derived populations, and myeloid-derived suppressor cells can limit T-cell priming and effector function. As Deng et al. showed through pre- and post-chemotherapy atlas analysis, neoadjuvant chemotherapy can reshape this immune landscape (7), but cannot reliably transform every lesion into an inflammatory, checkpoint-treatment-sensitive tumor. He et al. further confirmed at single-cell resolution that neoadjuvant chemotherapy alters but does not eliminate the immunosuppressive microenvironment (8). A comparative canine study by Regan et al. showed that losartan combined with the kinase inhibitor toceranib produced significant clinical benefit in dogs with metastatic osteosarcoma by blocking monocyte recruitment, providing translational evidence for targeting the myeloid circuit (9).

**Figure 1:**
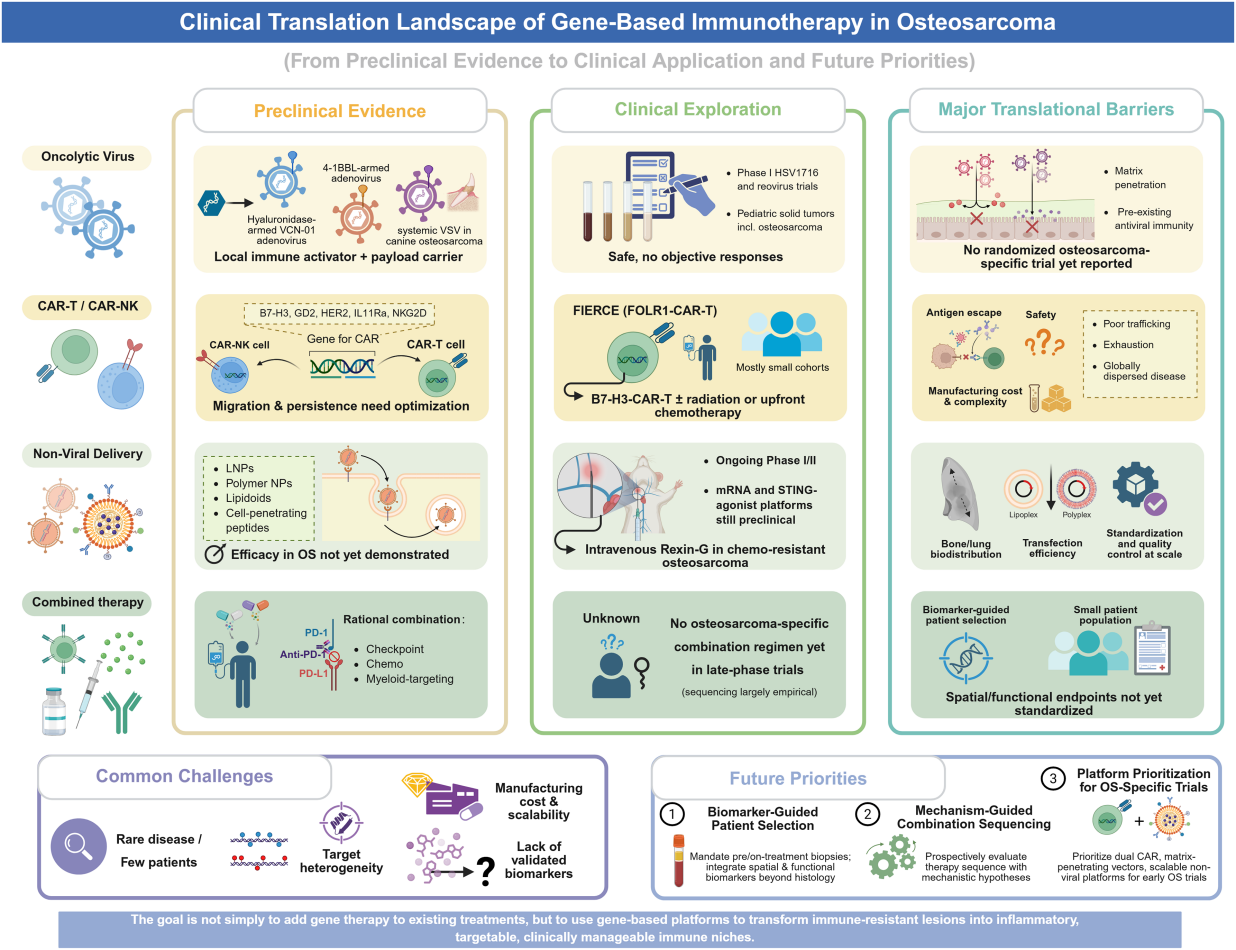
Gene-based immunotherapy reprograms the osteosarcoma microenvironment from an immune-resistant to an immune-responsive state. Before intervention, the primary bone lesion is characterized by an osteoid/mineralized matrix barrier, abnormal vasculature, weak antigen presentation, limited CD8^+^ T-cell trafficking, and immunosuppressive myeloid populations, including tumor-associated macrophages and myeloid-derived suppressor cells. The lung metastatic niche represents a distinct microenvironment with systemic delivery requirements and variable myeloid suppression. Gene-based approaches—including oncolytic viruses, cytokine-armed vectors, CAR-T/CAR-NK cells, and non-viral delivery systems—may promote tumor-antigen release, dendritic-cell priming, cGAS–STING-associated innate sensing, chemokine-guided immune-cell trafficking, and cytotoxic lymphocyte activity. The schematic illustrates proposed mechanisms of immune reprogramming and does not imply that all pathways have been clinically validated in osteosarcoma. Warm red and brown tones denote immune-resistant components and suppressive signals, whereas blue and green tones denote immune-responsive components and therapeutic immune activation. CAR, chimeric antigen receptor; cGAS, cyclic GMP–AMP synthase; DAMP, damage-associated molecular pattern; IFN, interferon; LNP, lipid nanoparticle; MDSC, myeloid-derived suppressor cell; ROS, reactive oxygen species; STING, stimulator of interferon genes; TAM, tumor-associated macrophage.

Conventional immunotherapy is also limited by other barriers. Osteosarcoma-associated antigens are heterogeneous and may be poorly expressed. Cytotoxic T cells may have difficulty trafficking into dense osteoid or mineralized tumor regions. NK cell activity can be limited by inhibitory cytokines and abnormal angiogenesis. Lung metastases can establish an immune microenvironment distinct from that of the primary bone lesion. Consequently, PD-1 blockade alone may lack a sufficiently active pre-existing immune response to amplify. Gene-based strategies may instead establish the immunological conditions required for treatment response: oncolytic vectors can promote tumor-antigen release and induce innate immune sensing; armed vectors can deliver payloads that are too toxic or too short-lived for systemic administration; and engineered cells can redirect recognition to specific targets. The key issue is not whether gene therapy can kill osteosarcoma cells in isolation, but whether it can transform resistant lesions into immune-responsive niches (**Figure 1**). A key knowledge gap is that no prospectively validated non-invasive marker currently distinguishes among immune-excluded, immune-desert, and transiently inflamed osteosarcoma lesions, and without a biopsy this state cannot be determined in practice, which limits rational, upfront selection of gene-based immunotherapy platforms.

## 3 Oncolytic vectors as *in situ* immune activators

Oncolytic vectors have a dual role, which is particularly relevant to osteosarcoma. They can directly lyse tumor cells, but their broader value may lie in transforming tumor tissue into sites of *in situ* immune activation. The oncolytic process releases tumor antigens, damage-associated molecular patterns, viral pathogen-associated signals, and inflammatory cytokines. These events can promote dendritic-cell activation, type I interferon signaling, cross-presentation, and the activation of T and NK cells (10, 11). For immune-resistant tumors, this vaccine-like function may be more important than the proportion of cells infected at a given time point.

Osteosarcoma-specific evidence remains limited but is biologically informative; osteosarcoma-related reviews also summarize major vector classes and delivery barriers (12, 13). Martinez-Velez et al. demonstrated significant antitumor activity of the oncolytic adenovirus VCN-01, which encodes hyaluronidase to degrade the extracellular matrix and improve viral spread, in a pediatric osteosarcoma model by overcoming the matrix barrier that limits conventional viral penetration (14). The same team subsequently showed that 4-1BBL-armed oncolytic adenovirus produced antitumor effects and durable immune memory in a pediatric osteosarcoma model, suggesting that the importance of payload design extends beyond the oncolytic scaffold itself (15). Makielski et al. reported that neoadjuvant systemic vesicular stomatitis virus was safe in dogs with naturally occurring osteosarcoma and may enhance long-term survival, providing a comparative model for systemic oncolytic delivery that more closely reflects the biology of human osteosarcoma than conventional rodent models (16). An *in vitro* osteosarcoma study of an anti-PD-1 sdAb-armed oncolytic virus demonstrated that vectors can be designed to carry locally delivered checkpoint blockade (17).

Clinically, however, osteosarcoma-specific evidence for oncolytic virotherapy remains limited to early-phase feasibility studies: a phase I trial of intratumoral HSV1716 in children with relapsed extracranial solid tumors, including osteosarcoma, demonstrated safety and evidence of viral replication but no objective tumor responses (18), and a Children’s Oncology Group phase I trial of intravenous reovirus in pediatric sarcomas showed a similar pattern of acceptable safety without objective tumor regression (19); no osteosarcoma-restricted oncolytic-virus trial has yet produced randomized or biomarker-stratified efficacy data or reported an osteosarcoma-specific efficacy endpoint. **Table 1** compares the major oncolytic viral platforms explored for osteosarcoma and related pediatric solid tumors in terms of their advantages and limitations.

**Table 1:** Comparison of major oncolytic viral platforms explored for osteosarcoma and related pediatric solid tumors.

Platform	Genome/cargo capacity	Delivery route	Key advantages	Key limitations	Osteosarcoma-specific evidence/stage
Adenovirus (e.g., VCN-01, 4-1BBL-armed)	dsDNA; ~7–8 kb; non-integrating	Intratumoral; intravenous delivery evaluated preclinically	Large transgene capacity; can be armed with matrix-degrading enzymes (hyaluronidase) to overcome the osteoid barrier; well-characterized platform biology and engineering	Pre-existing neutralizing immunity common; mainly local rather than systemic reach	Pediatric osteosarcoma xenograft models (14, 15); preclinical
Herpes simplex virus (HSV-1, e.g., HSV1716)	dsDNA; very large capacity; engineered lytic/oncolytic platform	Intratumoral injection	Large payload capacity; extensive pediatric intratumoral safety experience; strong local immune activation	Mainly local administration; limited penetration of dense matrix; theoretical latency/reactivation risk	Phase I pediatric trial including osteosarcoma: safe, no objective responses (18); clinical (phase I)
Vaccinia virus	dsDNA; >25 kb capacity	Intratumoral or intravenous (relatively complement-resistant)	Systemic delivery feasible; rapid replication; strong innate activation; cytokine arming with IL-12 showed an improved preclinical safety profile (20)	Prior vaccination history affects immunity; systemic cytokine toxicity risk when armed; shedding risk	Tested in osteosarcoma-specific preclinical models, including SIRPα-Fc-armed vaccinia virus in an immunocompetent murine model (21); no osteosarcoma-specific clinical evidence; preclinical
Vesicular stomatitis virus (VSV)	Negative-sense RNA; moderate capacity; rapid replication	Systemic (intravenous) feasible	Fast onset; low human seroprevalence; systemic delivery well tolerated in large-animal studies	Neurotoxicity risk with wild-type strains (mitigated by IFN-sensitizing engineering); less clinical experience overall	Safe, associated with improved survival in canine osteosarcoma (16); preclinical (large-animal)

dsDNA, double-stranded DNA; HSV, herpes simplex virus; IFN, interferon; VSV, vesicular stomatitis virus.

Armed vectors extend this concept. IL-12-armed vaccinia virus demonstrates that cytokine payloads can be localized to enhance immune activation while attempting to reduce systemic toxicity (20). Osteosarcoma-specific preclinical evidence also includes SIRPα-Fc-armed vaccinia virus, which delayed tumor progression and improved survival in an immunocompetent murine model (21). The GM-CSF-expressing talimogene laherparepvec demonstrated that local oncolytic immunotherapy can be clinically active, although evidence from melanoma cannot be directly extrapolated to osteosarcoma (22). Cytokine-armed herpes simplex virus vectors further support localized immune activation, although this evidence remains largely extrapolative for osteosarcoma (23). For osteosarcoma, payload selection should be matched to the dominant immune deficit: chemokines may be useful when effector cells cannot enter the tumor, whereas IL-12 or co-stimulatory ligands may be more relevant when dendritic-cell priming and activation are weak. The most logical role of oncolytic vectors in osteosarcoma is not as stand-alone lytic agents, but as programmable local immune activators that can provide a biological rationale for subsequent checkpoint blockade or cell therapy. A key knowledge gap is that with the exception of small, single-arm pediatric safety trials, no oncolytic vector has been tested against a defined osteosarcoma biomarker hypothesis (for example, baseline myeloid infiltration or antiviral immune status), so it remains unknown which patients are most likely to benefit.

## 4 Engineered immune cells for osteosarcoma

Engineered immune cells address a different limitation: endogenous lymphocytes may not be able to recognize osteosarcoma strongly enough or persist in the suppressive niche. CAR-T cells are the most mature platform. Ahmed et al. reported the first clinical trial of HER2-specific CAR-T cells for HER2-positive sarcoma, treating 19 patients, including osteosarcoma cases, with dose escalation to 1 × 10^8 cells/m². Treatment was feasible and well tolerated, with no dose-limiting toxicities; 4 of the 17 evaluable patients achieved disease stabilization for 12 weeks to 14 months, but no objective response was observed under RECIST criteria (24). Earlier work by the same team showed that genetic modification of T cells can overcome the low antigen density in osteosarcoma, which is an important proof of concept for tumors with weak target expression (25).

Target selection is also evolving. IL11Rα-guided T cells induced regression of established osteosarcoma lung metastases in preclinical models (26). NKG2D-CAR memory T cells may partly mitigate dependence on a single conventional tumor antigen by recognizing stress-induced ligands expressed by osteosarcoma cells (27). GD2-guided CAR-T cells with low-dose doxorubicin have shown preclinical synergy, suggesting that appropriately dosed chemotherapy may sensitize tumor cells to engineered immunotherapy without simply increasing toxicity (28). B7-H3 has attracted particular attention because it is widely expressed in pediatric solid tumors. Majzner et al. demonstrated potent preclinical activity of B7-H3 CAR-T cells against pediatric solid tumors and brain tumors (29), and Zhang et al. subsequently demonstrated its osteosarcoma-specific activity *in vitro* and *in vivo* (30).

A central limitation is that stronger antigen recognition does not necessarily translate into clinical efficacy in osteosarcoma. Antigen heterogeneity and variable antigen density can lead to escape, poor trafficking can prevent effector cells from reaching the tumor, and T-cell exhaustion, suppressive myeloid cells, hypoxia, and dense extracellular matrix can reduce persistence and cytotoxicity. These barriers may differ between a primary mineralized lesion and multifocal lung metastases, where systemic trafficking and lesion-to-lesion immune heterogeneity become especially important. Accordingly, recent engineering strategies must address more than antigen-specific killing. In pediatric sarcoma models, Lake et al. showed that redirecting B7-H3 CAR-T cells to the IL-8 gradient improved homing and antitumor activity (31); Talbot et al. obtained similar results by redirecting B7-H3 CAR-T cells to chemokines expressed in osteosarcoma (32). This shifts the design goal from antigen recognition alone to coordinated homing, persistence, and resistance to local inhibition. B7-H3 is promising, but it is not a strictly tumor-restricted antigen; normal-tissue expression and interpatient variation require careful safety assessment (29). Suicide switches, transient mRNA expression, and tunable or logic-gated receptors are therefore important safeguards when target selectivity is imperfect.

CAR-NK cells and innate-like engineered cells may offer complementary advantages. NK cells can recognize stress signals and may have different safety profiles, although persistence remains a challenge. Kaczanowska et al. characterized the immune determinants of GD2 CAR-T amplification in patients with solid tumors, revealing how host and tumor background shape cell-therapy behavior that preclinical models did not adequately predict (33). Fowler et al. demonstrated that engineered γδ T cells delivering payloads exhibited enhanced cytotoxicity, persistence, and efficacy in preclinical osteosarcoma models (34). mRNA-engineered EphA2 CAR-NK cells demonstrated preclinical activity in pediatric sarcoma models, including osteosarcoma, supporting transient, non-integrative engineering as a flexible strategy (35). A review of NK cells for pediatric solid tumors highlights that migration, persistence, and manufacturing remain major obstacles (36).

TCR-T and neoantigen-directed strategies may expand the target space beyond surface antigens, but they are currently exploratory rather than near-term clinical options for most patients with osteosarcoma. Their feasibility depends on HLA restriction, reliable presentation of the target peptide, identification of shared rather than purely private neoantigens, and sufficient clonal conservation across primary and metastatic lesions. In a genetically heterogeneous tumor with variable antigen presentation, individualized neoantigen discovery may be slow and resource-intensive for pediatric or adolescent patients with progressive disease. These constraints do not negate the value of TCR-based approaches, but they suggest that such approaches should follow more mature surface-targeted and delivery strategies in the current translational sequence (37, 38).

Resistance to CAR-T therapy in osteosarcoma is best understood as arising from several distinct, only partially overlapping mechanisms rather than a single failure mode. Antigen-level resistance includes both pre-existing heterogeneity in target expression across and within lesions and acquired antigen loss or downregulation under selective CAR-T pressure. Trafficking-level resistance reflects the difficulty of moving through dense osteoid and mineralized matrix and the absence of tumor-directed chemokine gradients, which chemokine-receptor engineering partially addresses (31, 32). Microenvironment-level resistance includes myeloid-derived suppressor cells and tumor-associated macrophages that inhibit T-cell function locally, TGF-β and other immunosuppressive cytokines, hypoxia, and nutrient competition within the tumor bed. Cell-intrinsic resistance includes progressive T-cell exhaustion and loss of persistence after repeated antigen encounter. Because these mechanisms operate at different levels, no single engineering fix is likely to be sufficient, and resistance should be characterized mechanistically in future osteosarcoma trials through paired pre- and post-treatment biopsies rather than assumed to be uniform across patients (39).

Dual-target and multi-target CAR strategies are a rational response to antigen-level resistance in particular. Bispecific or tandem CARs recognizing two osteosarcoma-associated antigens simultaneously (for example, B7-H3 combined with GD2 or HER2) can reduce the probability of complete antigen escape, since a tumor cell would need to lose both targets rather than one to evade recognition; AND-gated or OR-gated logic-controlled CARs can additionally improve on-tumor selectivity when neither antigen alone is sufficiently tumor-restricted. These potential advantages are offset by greater construct complexity, potentially reduced avidity for either target compared with a monospecific CAR, and more demanding manufacturing and release testing. For osteosarcoma, where no single surface antigen is both highly restricted and universally expressed, dual-targeting is conceptually attractive, but has so far been tested mainly in preclinical models or in other solid tumors, and prospective osteosarcoma-specific comparisons between single- and dual-target constructs are not yet available (39–41).

## 5 Gene delivery systems and immune-modulating payloads

Gene-based immunotherapy depends as much on delivery as on the payload. Viral systems differ in cargo capacity, tropism, expression duration, immunogenicity, and integration risk. Non-viral systems, including lipid nanoparticles, polymer nanoparticles, exosome-based delivery, hydrogels, and local biomaterials, offer greater design flexibility but often struggle with biodistribution, transfection efficiency, and tissue penetration. **Table 2** summarizes these trade-offs between viral and non-viral gene delivery systems in more detail.

**Table 2:** Comparison of viral versus non-viral gene delivery systems for osteosarcoma immunotherapy [sources (10, 11, 42–49)].

Feature	Viral vectors (adenovirus, HSV, vaccinia, VSV, retro-/lentivirus)	Non-viral systems (LNPs, polymer nanoparticles, exosomes, hydrogels)
Cargo capacity	Variable, approximately 4 to >30 kb depending on platform	Platform-dependent; commonly used for mRNA, siRNA/oligonucleotides, and plasmid DNA
Expression duration	Highly vector-dependent: transient for many non-integrating or oncolytic vectors, but potentially prolonged for integrating vectors	Typically transient; favorable when permanent integration is undesired
Immunogenicity	Can be substantial (anti-vector immunity, pre-existing neutralizing antibodies)	Generally lower, but can still trigger innate immune responses
Integration/insertional risk	Present for integrating vectors (retro-/lentivirus); minimal for adenovirus, HSV, vaccinia, VSV	Minimal to none for most platforms (LNPs, polymer nanoparticles)
Manufacturing complexity & cost	High; vector-specific good manufacturing practice production and batch-to-batch variability	Potentially more standardized and scalable, but cold-chain logistics and quality control remain demanding
Bone/tumor tissue penetration	Limited by dense osteoid and mineralized matrix; local injection often required	Similarly limited; nanoparticle size and surface chemistry strongly affect penetration
Repeat dosing feasibility	Constrained by anti-vector neutralizing immunity after first dose	Generally more feasible; fewer neutralizing-immunity concerns
Regulatory pathway maturity	Multiple approved oncology products (e.g., T-VEC) provide precedent	Fewer approved oncology precedents; mRNA-LNP platforms benefit from COVID-19 vaccine experience

HSV, herpes simplex virus; LNP, lipid nanoparticle; mRNA, messenger RNA; siRNA, small interfering RNA; T-VEC, talimogene laherparepvec; VSV, vesicular stomatitis virus.

These barriers differ meaningfully between the primary bone lesion and lung metastases, and delivery strategy should be matched accordingly. In the primary osteoid and mineralized bone lesion, dense extracellular matrix, sparse and abnormal vasculature, and low interstitial fluid flow limit both viral particle spread and nanoparticle penetration; local or intratumoral administration, matrix-degrading enzyme arming [e.g., hyaluronidase-expressing adenovirus (14)], and image-guided injection are therefore more relevant than systemic delivery for bone-confined disease. In contrast, pulmonary micrometastases, the dominant site of lethal osteosarcoma relapse, are typically small, multifocal, and better vascularized than the primary tumor, favoring systemic intravenous delivery, but they also present a more heterogeneous and rapidly evolving immune microenvironment across lesions, and systemic vector or nanoparticle exposure raises distinct concerns about pulmonary toxicity and off-target biodistribution to the liver and spleen. Few studies have directly compared delivery performance between primary bone and lung metastatic sites within the same osteosarcoma model, which remains an important gap for rational vector selection.

These delivery constraints are particularly pronounced in osteosarcoma. Primary bone tumors contain osteoid, mineralized matrix, abnormal vasculature, and necrotic regions, whereas pulmonary metastases may form multiple spatially dispersed deposits. Chawla et al. tested intravenous Rexin-G, a targeted retroviral vector carrying a cytocidal cyclin G1 construct, in chemotherapy-resistant sarcoma and osteosarcoma patients, providing one of the earliest clinical examples of systemic gene delivery in this disease context; however, its payload is cytocidal rather than directly immunomodulatory (42). More recent non-viral work has explored biodegradable carbon dioxide-derived vectors for osteosarcoma gene delivery (43) and multifunctional nanoplatforms combining gene delivery with NIR-II photothermal therapy (44). Nanoparticle delivery of miRNAs has also been reviewed as a strategy to regulate osteosarcoma biology, although immune reprogramming applications remain less mature than tumor-intrinsic applications (45).

The range of potential payloads is broad, but an immunotherapy-focused review should distinguish tumor-intrinsic delivery from immune-modulating delivery. Tumor-cell-directed RNA interference, miRNA replacement, or cytotoxic-gene approaches may directly impair malignant cells, whereas cytokines, chemokines, checkpoint-blocking molecules, co-stimulatory ligands, tumor antigens, STING or TLR agonists, and transient mRNA constructs for cell engineering are intended to alter the immune compartment or stromal niche. Advances in mRNA non-viral delivery and lipid nanoparticles make transient immune modulation increasingly plausible, especially when permanent integration is not desired (46, 47). Exosome-based systems may offer biocompatibility advantages, but loading efficiency, standardization, and scalable manufacturing remain unresolved (48). Nanoparticle-mediated STING-agonist delivery illustrates how packaging can improve tumor localization and reduce systemic exposure (49). For osteosarcoma, the intended target compartment must be explicit: a mineralized primary lesion, a micrometastatic lung deposit, a suppressive myeloid niche, and a bulky recurrent tumor may each require different vector kinetics, immune payloads, and safety controls.

## 6 Combination strategies and translational barriers

The clinical trial landscape for gene-based immunotherapy in osteosarcoma remains dominated by preclinical and early-phase studies, and osteosarcoma-specific trials remain scarce relative to the broader sarcoma and pediatric solid-tumor literature. Early-phase pediatric studies have established the feasibility of intratumoral and intravenous oncolytic virotherapy in extracranial solid tumors that included osteosarcoma: a phase I trial of the oncolytic herpes simplex virus HSV1716 delivered by image-guided intratumoral injection was safe and showed evidence of viral replication and immune activation, although no objective responses were observed (18), and a Children’s Oncology Group phase I trial of intravenous reovirus (Reolysin) in relapsed or refractory extracranial solid tumors, including several sarcoma histologies, likewise demonstrated safety without objective tumor regression (19). These trials establish feasibility rather than efficacy. ClinicalTrials.gov registry records were rechecked on July 19, 2026. No active or recently completed oncolytic-virus trial restricted to osteosarcoma has produced randomized or biomarker-stratified efficacy data or reported an osteosarcoma-specific efficacy endpoint; available oncolytic-virus evidence therefore comes mainly from pediatric/solid-tumor trials that include osteosarcoma or from broader solid-tumor OV studies. Two additional registry records of recombinant oncolytic HSV-1 R130 in advanced or relapsed/refractory bone and soft-tissue tumors list osteosarcoma among the eligible conditions (NCT06171282 and NCT05851456). ClinicalTrials.gov currently lists the overall recruitment status of both records as “Unknown,” although their last known status was “Recruiting” (last verified in December 2023 for NCT06171282 and April 2023 for NCT05851456). Neither study is restricted to osteosarcoma; they should therefore be interpreted as mixed-cohort studies with outdated registry status rather than confirmed actively recruiting osteosarcoma-specific trials.

By contrast, osteosarcoma-directed CAR-T trials are now emerging. The FIERCe trial (FOLR1 immune effector cell therapy against advanced osteosarcoma; NCT07227571) is recruiting patients with advanced refractory or recurrent/progressive osteosarcoma to test FH-FOLR1 ST CAR-T cells. B7-H3-directed CAR-T approaches include a pediatric solid-tumor phase I trial that includes osteosarcoma (NCT04897321), a recurrent/refractory pediatric solid-tumor study including osteosarcoma that is active but not recruiting (NCT04483778), and a phase II study testing consolidative B7-H3 CAR-T cells after upfront MAP chemotherapy in newly diagnosed high-risk pediatric osteosarcoma (NCT07428993). A recent systematic analysis of the osteosarcoma trial landscape found that immuno-oncology approaches now account for a large share of novel-agent trials, that more than 90% of all osteosarcoma trials remain in phase I/II, and that late-phase, biomarker-stratified trials are conspicuously rare (50). Taken together, the trial landscape confirms that gene-based immunotherapy in osteosarcoma is still at the stage of establishing safety and biological proof of concept, and that the field’s central unmet need is a small number of well-designed, mechanism-linked phase II trials rather than additional preclinical platforms.

Combination strategies are likely to be necessary because immune resistance in osteosarcoma is multifactorial. Oncolytic vectors can provide antigen release and innate immune activation, but checkpoint blockade may still be necessary if newly primed T cells become exhausted. Engineered immune cells may require myeloid reprogramming, vascular normalization, or chemokine-receptor matching to reach the tumor. Gene therapy can also be paired with chemotherapy or radiotherapy when cytotoxic therapy increases antigen release or enhances sensitivity to immune killing (51). Combination design should be guided by lesion biology rather than empirical convenience. Immune-cold primary tumors may be suitable for oncolytic or innate immune agonist platforms. Metastases with high B7-H3 expression but poor T-cell trafficking may require engineered CAR-T cells with homing receptors. A myeloid-rich lung metastatic niche may require macrophage or monocyte modulation before cell therapy. The order of administration is equally important: administering the vector before cell therapy may increase antigen release and chemokine production, while administering the vector after cell infusion may support persistence but may also increase inflammatory toxicity.

Clinical translation nevertheless remains challenging. The patient population is small, pediatric development requires stringent safety standards, and endpoints for metastatic or recurrent disease are difficult to standardize. Vector neutralization, insertion risk, cytokine toxicity, neurotoxicity, production costs, release testing, and regulatory complexity must all be addressed as early as possible. The heterogeneity between primary tumors and metastases also means that a single biopsy may not capture the truly relevant immune barriers. Current bone tumor guidelines emphasize the need for multidisciplinary treatment and clinical trial enrollment, especially for recurrent or refractory disease (52). Biomarkers should be spatial and functional. Target antigen density, myeloid infiltration, interferon signaling, chemokine gradients, vascular dysfunction, lung metastasis immune status, and circulating immune markers may be more informative than bulk PD-L1 expression alone. Pharmacodynamic endpoints should be included in trials to show whether the gene-based platform has truly reprogrammed the target immune niche. Paired biopsies, image-guided sampling, circulating tumor DNA dynamics, and immune cell atlases can help differentiate between biological failure and inadequate delivery. This distinction is important: a negative trial may reflect an inadequate dose, an incorrect route, insufficient intratumoral exposure, or inappropriate patient selection, rather than a failure of the entire platform category. However, much of this combination logic is inferred from adult solid-tumor and melanoma data rather than demonstrated directly in osteosarcoma, and prospective validation in osteosarcoma-specific models and trials is still needed before any sequencing strategy can be considered established.

Candidate markers for prospective patient selection include tumor surface antigen density, such as HER2, GD2, B7-H3, and IL11Rα, measured in diagnostic biopsy or metastasectomy specimens; the baseline myeloid-to-lymphoid ratio and macrophage density; interferon-stimulated gene expression as a proxy for baseline inflammation; and circulating markers such as ctDNA burden or myeloid-derived suppressor cell frequency. However, none of these markers has been prospectively validated as a predictive biomarker in osteosarcoma, and most current trials continue to enroll biomarker-unselected patients. Incorporating pretreatment biopsies and biomarker assessments into trial design should therefore be regarded as a near-term priority, because gene-based immunotherapy is unlikely to benefit an unselected osteosarcoma population.

**Figure 2:**
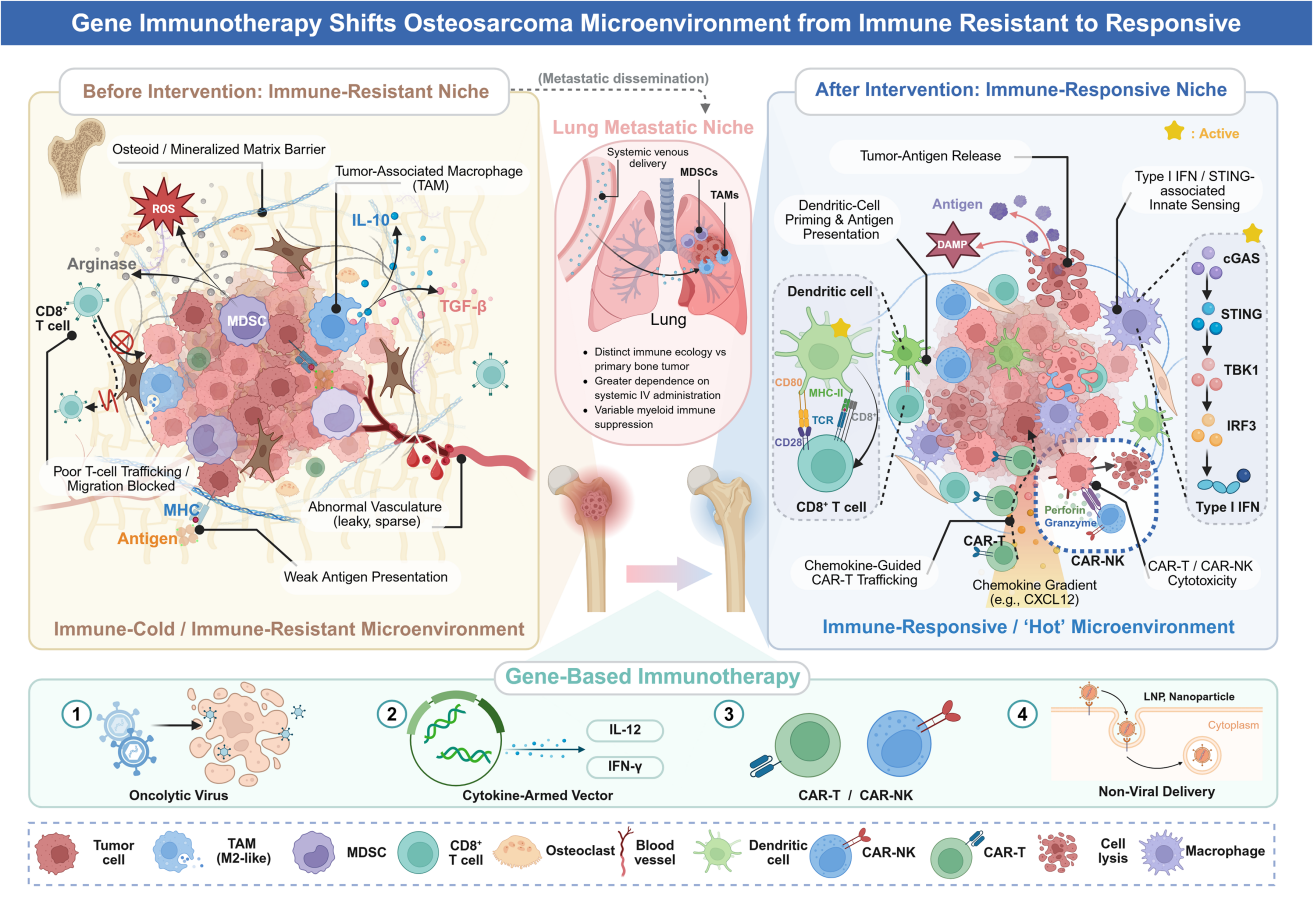
Clinical translation landscape of gene-based immunotherapy in osteosarcoma, from preclinical evidence to clinical application and future priorities. For each platform class (oncolytic virus, CAR-T/CAR-NK, non-viral delivery, and combination therapy), the panel summarizes representative preclinical evidence, corresponding clinical exploration (including early-phase feasibility trials, the FIERCe FOLR1-CAR-T trial, B7-H3-CAR-T programs, and Rexin-G as a systemic cytocidal gene-delivery precedent rather than an immune-modulating therapy), and the major translational barriers specific to that platform (matrix penetration, antiviral immunity, antigen escape, trafficking and persistence, manufacturing complexity, biodistribution, and lack of validated biomarkers). Common cross-platform challenges (small rare-disease patient populations, target heterogeneity, and absent validated biomarkers) and three proposed future priorities—biomarker-guided patient selection, mechanism-guided combination sequencing, and platform prioritization for osteosarcoma-specific trials—are shown at the bottom. The overarching message is that gene-based platforms should be used not simply as an addition to existing therapy, but to transform immune-resistant osteosarcoma lesions into clinically manageable immune niches.

Manufacturing feasibility also represents an important translational barrier. Autologous CAR-T and CAR-NK products require individualized vector production, cell expansion, and release testing, whereas allogeneic or off-the-shelf products may improve scalability but introduce concerns regarding immune rejection and limited persistence. Viral-vector production is similarly costly and platform-specific, while non-viral systems may be easier to standardize and scale but still require rigorous quality control and cold-chain infrastructure. Manufacturing cost and scalability should therefore be considered during early platform selection rather than only after clinical efficacy has been demonstrated (41, 47, 48). Taken together, **Table 3** compares the translational maturity of the major gene-based immunotherapy strategies, whereas **Figure 2** integrates these platforms into a translational pipeline from preclinical validation to clinical application and highlights the principal challenges at each stage.

**Table 3:** Representative gene-based immunotherapy strategies for osteosarcoma and their translational maturity.

Strategy	Representative platforms/evidence base	Main immune mechanism	Potential value in osteosarcoma	Key limitations	Stage of development
Oncolytic vectors	VCN-01 and 4-1BBL-armed adenovirus (osteosarcoma preclinical); HSV1716 and reovirus (mixed pediatric phase I); VSV (canine comparative oncology) (14–16, 18, 19)	Tumor lysis; immunogenic cell death; antigen release; innate immune activation	May convert immune-cold lesions into inflamed, vaccine-like sites	Delivery; antiviral immunity; tumor heterogeneity; pediatric safety	Osteosarcoma preclinical; early clinical feasibility in mixed pediatric cohorts
Cytokine-armed vectors	4-1BBL-armed adenovirus and anti-PD-1 sdAb virus (osteosarcoma preclinical); IL-12/GM-CSF vectors (mostly other tumors) (15, 17, 20–23)	Local dendritic-cell activation; T-cell priming; NK-cell support	May amplify local immunity while limiting systemic cytokine exposure	Dose control; local toxicity; expression duration; regulation	Osteosarcoma preclinical; early clinical evidence mainly extrapolated
CAR-T cells	HER2 CAR-T (phase I sarcoma including osteosarcoma); GD2 and B7-H3 CAR-T (osteosarcoma preclinical and early trials) (24, 28–32)	Redirected antigen-specific T-cell cytotoxicity	May target resistant cells expressing defined surface antigens	Antigen escape; poor infiltration; exhaustion; off-tumor toxicity	Preclinical to early clinical (phase I/II)
CAR-NK and innate-like cells	mRNA-engineered EphA2 CAR-NK (sarcoma); engineered γδ T cells (osteosarcoma preclinical) (34–36)	CAR recognition plus innate cytotoxicity or payload delivery	May suit heterogeneous or low-MHC immune niches	Persistence; expansion; durability; manufacturing	Preclinical to early clinical; little osteosarcoma-specific clinical evidence
TCR or neoantigen therapy	TCR-engineered cells and NeoTCR approaches (mostly other tumors) (37, 38)	Recognition of intracellular antigens presented by HLA molecules	May expand target space beyond surface antigens	HLA restriction; antigen discovery; clonal heterogeneity; variable presentation	Exploratory; no established osteosarcoma clinical path
Non-viral delivery	LNPs, polymer nanoparticles, exosomes, hydrogels, and STING/TLR or RNA payloads (43–49)	Transient delivery of mRNA, siRNA, miRNA, or innate immune cargo	Flexible, repeatable, potentially non-integrating immune modulation	Bone penetration; biodistribution; stability; quality control	Preclinical; osteosarcoma evidence remains mainly tumor-intrinsic
Combination platforms	Vectors plus checkpoint blockade, chemotherapy, radiotherapy, anti-angiogenic, or myeloid-targeting therapy (51)	Matches gene-based activation to the dominant immune barrier	May address multifactorial resistance	Toxicity; sequencing; biomarker selection; trial complexity	Preclinical to early clinical; mostly extrapolated from broader solid tumors

Evidence labels distinguish osteosarcoma-specific studies, comparative oncology, mixed pediatric solid-tumor cohorts, and extrapolated evidence from other tumor types.

## 7 Conclusion

Gene-based immunotherapy in osteosarcoma is best understood as a strategy for immune-niche reprogramming. The main challenge is not merely the difficulty in killing osteosarcoma cells, but the fact that resistant lesions often exhibit antigenic heterogeneity, myeloid suppression, poor lymphocyte migration, physical matrix barriers, and metastatic immune adaptation. Gene-based platforms are attractive because they can be programmed to address multiple barriers simultaneously.

Oncolytic vectors can serve as local immune activators and payload carriers. Engineered T cells, NK cells, γδ T cells, and TCR-engineered approaches can enforce recognition, but migration, persistence, and resistance to suppression must also be optimized. Non-viral systems may allow reproducible, transient delivery of immunomodulatory payloads, although their delivery performance in osteosarcoma requires direct validation. Future advances will depend on rational vector selection, payload design, spatial biomarkers, and mechanism-guided combination therapies. In practical terms, immune-cold lesions with weak antigen release may be better candidates for oncolytic vectors or innate immune agonists; B7-H3-high tumors with poor T-cell trafficking may require CAR-T cells equipped with homing receptors; and lung metastases with pronounced myeloid suppression may require myeloid-targeting combinations before or alongside cell therapy. Clinical translation should proceed cautiously, especially in pediatric and adolescent patients.

Three priorities should anchor the next phase of clinical translation. First, trials should incorporate mandatory pre- and on-treatment biopsies or minimally invasive surrogates so that spatial and functional biomarkers, rather than histologic subtype alone, guide patient selection. Second, combination sequencing, including whether a vector is administered before or after cell therapy and whether it is paired with checkpoint blockade, chemotherapy, or myeloid-targeting agents, should be tested prospectively against a clear mechanistic hypothesis rather than empirically. Third, dual-target CAR constructs, matrix-penetrating armed vectors, and scalable non-viral delivery platforms should be prioritized for early-phase osteosarcoma-specific trials, since these directly address the antigen-escape, matrix, and manufacturing barriers identified throughout this review. The next step is not simply to add gene therapy to existing osteosarcoma treatment, but to use gene-based platforms to transform immune-resistant lesions into clinically manageable immune niches.
